# Reliable wrist PPG monitoring by mitigating poor skin sensor contact

**DOI:** 10.1038/s41598-025-31883-5

**Published:** 2025-12-17

**Authors:** Hung Manh Pham, Matthew Yiwen Ho, Yiming Zhang, Dimitris Spathis, Aaqib Saeed, Dong Ma

**Affiliations:** 1 School of Computing and Information Systems, Singapore Management University, Singapore, Singapore; 2 Department of Computer Science and Technology, University of Cambridge, Cambridge, UK; 3 Google, London, UK; 4 Department of Industrial Design, Eindhoven University of Technology, Eindhoven, The Netherlands

**Keywords:** PPG Transformation, Contact Pressure, Skin-Sensor Contact, Biomedical engineering, Health care

## Abstract

Photoplethysmography (PPG) is a widely used non-invasive technique for monitoring cardiovascular health and various physiological parameters on consumer and medical devices. While motion artifacts are well-known challenges in dynamic settings, *suboptimal skin-sensor contact in sedentary conditions* - *an important issue often overlooked in existing literature* - can distort PPG signal morphology, leading to the loss or shift of essential waveform features and therefore degrading sensing performance. In this work, we propose a deep learning-based framework that transforms Contact Pressure-distorted PPG signals into ones with the ideal morphology, known as CP-PPG. CP-PPG incorporates a well-crafted data processing pipeline and an adversarially trained deep generative model, together with a custom PPG-aware loss function. We validated CP-PPG through comprehensive evaluations, including 1) morphology transformation performance, 2) downstream physiological monitoring performance on public datasets, and 3) in-the-wild performance. Extensive experiments demonstrate substantial and consistent improvements in signal fidelity (Mean Absolute Error: 0.09, 40% improvement over the original signal) as well as downstream performance across all evaluations in Heart Rate (HR), Heart Rate Variability (HRV), Respiration Rate (RR), and Blood Pressure (BP) estimation (on average, 21% improvement in HR; 41-46% in HRV; 6% in RR; and 4-5% in BP). These findings highlight the critical importance of addressing skin-sensor contact issues for accurate and reliable PPG-based physiological monitoring. Our implementation is publicly available at: https://github.com/manhph2211/CP-PPG.

## Introduction

Photoplethysmography (PPG) is a non-invasive optical technique used to measure changes in blood volume in peripheral blood vessels. It functions by emitting light onto the skin using an LED and measuring the amount of light transmitted through (transmissive PPG) or reflected against (reflective PPG) the skin, which changes due to cardiac pulsations, with a photodetector. An ideal PPG waveform reflects complete cardiac cycles and contains all key features such as the systolic peak, dicrotic notch, and diastolic peak, all of which hold significant clinical importance. For example, PPG waveforms can be used to derive various physiological parameters, including heart rate (HR)^1–3^, heart rate variability (HRV)^4–7^, respiration rate (RR)^8,9^, blood pressure (BP)^10–13^, and blood oxygen saturation (SpO2)^14^, to detect infectious diseases^15^, as well as to provide valuable information about cardiovascular functions such as artery stiffness^16,17^, atrial fibrillation^18^, diabetes^19^, and chronic obstructive pulmonary disease (COPD)^20^. Furthermore, PPG signals have been shown to potentially enable applications such as biometric authentication^21^ and facial expression recognition^22^.

**Fig. 1:**
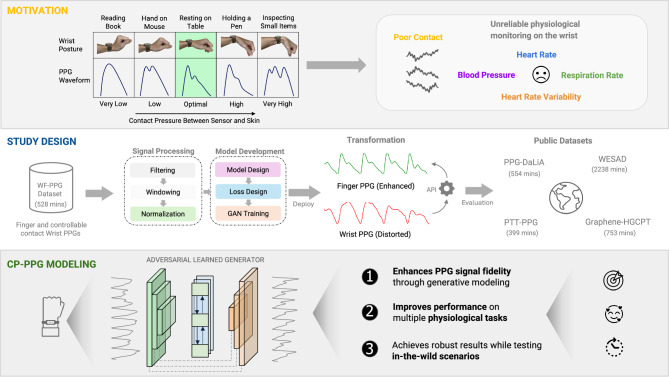
Study and experimental design. Poor skin-sensor contact can lead to significant distortion in PPG morphology. Our method successfully transforms distorted PPG signals from the wrist into reference PPG signals. Subsequently, we evaluate these enhanced signals through three comprehensive experiments with various datasets and physiological tasks.

With the advancement of mobile health, PPG has emerged as a leading technology for continuous health monitoring on wearable devices. For instance, many off-the-shelf smartwatches (e.g., Apple Watch^23^, Samsung Galaxy Watch^24^, and others) and even some recent earphones^25^ are equipped with PPG sensors for estimating HR, HRV, BP, and SpO2. Despite its successful adoption in commercial products, PPG remains an active research area due to its susceptibility to various real-world factors that can compromise its sensing performance. Prior research mainly focused on **motion artifacts**, defined as distortions of the PPG signal caused by user movement during physical activities such as walking and running^26–29^. Proposed solutions, such as adaptive filtering, accelerometer-based signal compensation, and deep learning algorithms, have shown promising results^30–33^.

Nevertheless, the sensing performance of PPG in **sedentary** scenarios has often been overlooked. For instance, as shown in Fig. 1 (Motivation), in smartwatch-based PPG sensing, individuals may adopt various wrist postures during different sedentary activities (e.g., reading), resulting in changes in contact pressure (CP) between the PPG sensor and human skin (i.e., tightness). Previous studies have shown that variations in CP can affect the accuracy of physiological parameter estimation through field experiments^34,35^, highlighting the importance of considering CP during estimation. *However, these studies have primarily focused on the influence of CP on signal amplitude (or signal-to-noise ratio) while 1) ignoring its impact on other key waveform features (e.g, presence of dicrotic notch, systolic/diastolic width) and 2) without proposing any solutions to mitigate these impacts.*

In our study, we observe that, in addition to signal amplitude, the **entire morphology** of the PPG waveform can be influenced by CP. Although these distortions are less extreme than those caused by motion, they prevent the extraction of more subtle yet important features for complex PPG applications. For example, certain wrist postures (e.g., hand on mouse) can cause temporal shifts in peak positions, which negatively impact HR and HRV estimation that depend on precise timing. Meanwhile, in some postures (e.g., reading a book and holding a pen), the dicrotic notch and diastolic peak may be diminished or even absent altogether. Ultimately, downstream applications relying on these features (e.g., BP estimation) may be rendered unfeasible even in seemingly ideal, motionless conditions. Following that, we analyzed the signal waveforms in four *public* PPG datasets (as introduced in the Evaluation Section) collected during sedentary conditions and found only 19.6% of the PPG cycles present the ideal morphology, highlighting the difficulty of acquiring ideal PPG morphology in practical scenarios.

Therefore, developing a robust solution to enhance the quality of PPG signals under suboptimal CP is essential. To address this, we propose a deep learning-based framework called CP-PPG, designed to restore the ideal PPG morphology, typically observed under optimal CP, from distorted signals measured under sedentary conditions with suboptimal CP. Specifically, CP-PPG 1) processes a first-of-its-kind public dataset that includes two synchronously measured PPG streams - one from the wrist and the other from the finger. The wrist PPG was exposed to various contact pressures to produce a diverse range of PPG morphologies, while the finger PPG was maintained at an optimal CP to obtain the ideal morphology; 2) incorporates a novel generative deep neural network (DNN) and trains it with a custom loss function incorporating PPG domain knowledge and adversarial training techniques. We evaluated CP-PPG through comprehensive experiments and demonstrated its superior performance in transforming distorted morphologies into forms more accurately representing physiological attributes on the custom testing data. Additionally, with experiments on four public PPG datasets and a longitudinal in-the-wild study, we showed that the trained model can generalize across various data, and transformed waveforms enable more accurate estimation of typical physiological metrics, including HR, HRV, BP, and RR. These experiments also highlight the generalizability of CP-PPG, positioning it as a valuable **plug-in API** for refining PPG morphology for more accurate and robust PPG sensing in unseen conditions. We summarize the contributions of this work as follows:

- To the best of our knowledge, we are the first to address the impact of contact pressure on PPG signal morphology and downstream physiological sensing applications.
- We developed a comprehensive data pipeline and a novel adversarial DNN that particularly leverages gating mechanisms and PPG domain knowledge for effective PPG morphology transformation.
- With extensive experiments on public datasets, and an in-the-wild longitudinal study, we demonstrated the effectiveness and generalizability of the proposed approach.

## Methodology

**Fig. 2:**
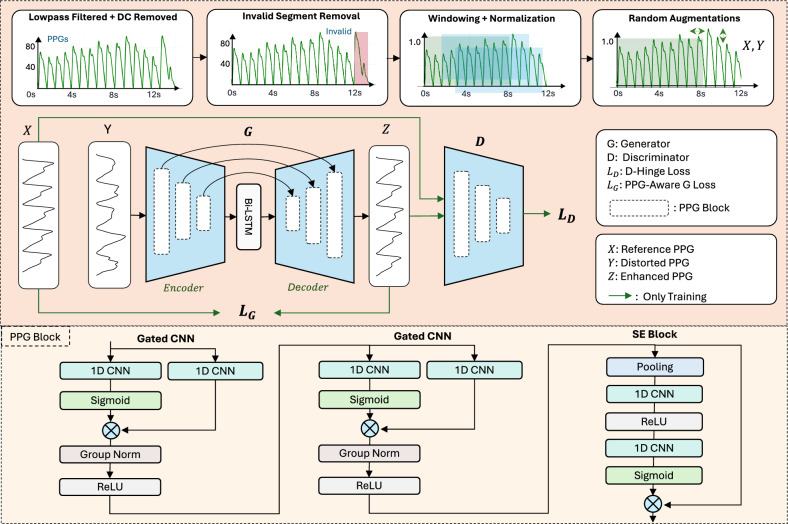
Illustration of CP-PPG framework. We begin with comprehensive data preprocessing to prepare the training data consisting of PPG pairs: distorted signals (*Y*) and their corresponding reference signals (*X*). We then train a lightweight U-shaped model comprising an encoder and decoder (each built with three specialized PPG blocks), optimized using adversarial training ($L_D$) and a custom loss function ($L_G$). Here, each PPG block is designed to extract critical features from the PPG signal by incorporating gated convolutional layers and an SE architecture.

We propose to restore ideal PPG waveforms from distorted ones using a deep learning-based framework. As depicted in Fig. 2, we first prepare a high-quality dataset for model training by devising a comprehensive signal processing pipeline. Subsequently, we introduced a novel deep neural network along with a set of training strategies to effectively facilitate the training process.

### Data preparation

Restoring the ideal PPG waveform using a deep learning-based approach requires a paired dataset, where the distorted waveform serves as the input and the ideal waveform as the label, for model training. However, most of the existing publicly available PPG datasets contain only a single-stream PPG signal collected from one location. There are a few datasets that collect both wrist and finger PPG simultaneously^36–38^, however, they are designed to address their specific downstream tasks (e.g., feature analysis^36^, vasoconstriction^37^, and mental workload^38^) without specifically concentrating on CP-induced distortions. Fortunately, a recent public dataset, WF-PPG^39^, provides distorted wrist PPG waveforms under varying CPs along with the corresponding ideal morphology (finger) recorded synchronously, which meets our requirements and is therefore selected for this study. Here, we choose the dataset’s most representative and well-collected 22 subjects (15 Male, 7 Female, mean age 24.01 ± 2.33). In total, we processed 528 minutes of data, which includes three simultaneously recorded signals for dealing with contact pressure variations: 1) wrist PPG signal subject to a full range of contact pressures, 2) ideal PPG signal, and 3) ECG signals. Here, the two PPG signals were sampled at 128 Hz, and the ECG signal at 130 Hz. For training and validation, we partitioned the 22 subjects into three separate sets (no overlapped subjects): training (13 subjects), validation (4 subjects), and testing (5 subjects).

In addition to conducting experiments using the WF-PPG dataset, we would further evaluate our model on other public datasets and, notably, on a self-collected in-the-wild dataset involving five recruited participants (see Evaluation section). The study was approved by the Institutional Review Board of the Singapore Management University (IRB approval number: IRB-23-207-A141-1224). Here, informed consent was obtained from all participants before their involvement in the study. All methods and experimental procedures were also conducted in accordance with the institutional guidelines and regulations.

### Signal processing

PPG signals are generally noisy, so to prepare them for model training or downstream evluation tasks, we developed a customized suite of signal processing techniques as follows:

*Lowpass fitering and DC removal: * We first removed high-frequency noise using a 5th-order Butterworth lowpass filter with a 10Hz cutoff^39–41^, and also eliminated the direct current (DC) component to retain only the meaningful pulsatile component (AC) of the signals, which reflects blood volume changes due to heartbeats^39,42^. These steps were applied to all datasets used in our study, except for WF-PPG, which had already undergone such preprocessing in its original release.

*Invalid segment removal: * Although participants in the WF-PPG dataset were instructed to remain still during each four-minute session, occasional wrist and finger movements were unavoidable. Consequently, some finger PPG waveforms deviate from the ideal morphology, and wrist PPG signals may be corrupted by motion artifacts. Additionally, inherent heartbeat variations, influenced by factors such as breathing, can cause deviations in PPG waveforms^43^. Given that the finger PPG serves as the ground truth for model training, it is crucial to ensure all finger PPG signals used for training exhibit the ideal morphology. Specifically, we first used peak detection to identify the peaks of each waveform, extracting individual heartbeat cycles and normalizing their amplitudes. Peak detection was used again on each cycle to locate the systolic peak, the dicrotic notch, and the diastolic peak. An ideal PPG waveform includes, from left to right, a prominent systolic peak (the maximum amplitude), a dicrotic notch, and a smaller diastolic peak (between 30%-90% of the maximum amplitude). Waveforms lacking these features, or containing extraneous features (e.g., more peaks or notches), were considered invalid. Corresponding segments in the wrist PPG signal were also eliminated to maintain synchronization. Consequently, finger PPG signals exhibit ideal morphology, while wrist PPG retains diverse patterns.

*Windowing: * Next, to facilitate the model training, we split the signals into windows using a sliding window technique. Specifically, windowing allows the continuous PPG signals to be split into fixed-length, model-compatible segments. Additionally, a sliding window allows better use of our dataset, allowing each waveform to occur within multiple windows at different positions to increase training data size. Following existing studies^44–46^, the default window length was designated as 8 seconds, with a 1.6-second sliding increment. We evaluate the impact of window length on the transformation performance in our ablation study (Table 2). After windowing, the wrist and finger PPG signals were converted to multiple instances, each comprising a pair of synchronized windows derived from the two signals.

*Normalization: * PPG signal amplitudes can vary greatly among individuals due to factors such as skin tone variations^47^, strengths of heartbeats^48^, and even within an individual owing to factors like contact pressure^49^. Since we aim to restore the morphology of the PPG signal, i.e., relative amplitudes within a window, we applied min-max normalization to scale each window between 0 and 1.

*Data augmentation: *Due to varying participant characteristics, behaviors (e.g., different frequencies and degrees of hand movements), and skin factors, the percentage of valid segments across individuals can be uncontrollably discrepant. Specifically, given the relatively small dataset size and the natural variability across subjects, certain waveform patterns may still be underrepresented, reducing their representation in the overall dataset and therefore the model’s ability to learn their patterns. Data augmentation addresses these issues by increasing more variants of these patterns, improving our model’s learning and performance. We balanced the dataset using data augmentation techniques, including random time warping, jittering, baseline drifting, and amplitude scaling^50^. Ultimately, we obtained over *4,600 eight-second [wrist PPG, finger PPG] window pairs* for subsequent model training and evaluation.

### Deep neural network design

To effectively transform the distorted PPG morphology into the ideal morphology, we propose a lightweight generative adversarial-based architecture as presented in Fig. 2. At a high level, it is composed of a generator (*G*) sub-network and a discriminator (*D*) sub-network, and was trained in an adversarial manner. The generator transforms the distorted wrist PPG signals into high-quality signals, while the discriminator functions as an auxiliary component, together with a custom loss function, further enhancing the generator’s capacity to generate better-fidelity samples.

#### Generator

For the generator model, we designed an enhanced autoencoder model with a fully convolutional encode–decode architecture and a Bi-directional Long Short-Term Memory layer (Bi-LSTM) introduced in the latent space. The encoder and decoder include three PPG blocks with skipping connections between corresponding blocks at different feature resolutions. Each PPG block starts with two sequences of Gated Convolutional Neural Network (Gated CNN) followed by a GroupNorm, a regular ReLU activation, and ends up with a Squeeze and Excitation (SE) Block. In the encoder, the number of channels initially starts at 1, and progressively increases to 32, 64, and finally 128, as the input signal passes through the convolutional layers within the PPG Blocks to get high-level features from the input. Conversely, the number of channels in the decoder gradually decreases, reverting to 1 as the desired enhanced signal. Meanwhile, the Bi-LSTM layer in the latent space enhances the model’s ability to capture temporal and long-range dependencies. It’s worth noting that this layer will double the number of output channels. Therefore, we incorporate a Dense layer to reduce it back to 128. In the following sections, we further detail important components of the generator model.

**Gated CNN block**. The Gated CNN block is inspired by the GRU layer’s gate design^51^, which can be used to retain necessary features while reducing unimportant input features. As shown in Fig. 2, our Gated CNNs are designed as a 1D CNN layer with a kernel size of 3, a stride of 1, and a padding of 1, adapting an additional gating branch. On one hand, the CNN layer aids the model in learning generalized features from dynamically varying PPG signals, allowing the encoder and decoder to capture useful temporal dependencies. On the other hand, the gating mechanism adjusts the flow of information through another 1D CNN layer using the Sigmoid function, which emphasizes and suppresses signal attributes based on their relevance to the learning context.

**Squeeze and excitation (SE) block**. Instead of assigning equal importance to all embedding channels as in traditional CNN layers, the SE block dynamically adjusts the weights of each channel based on its importance in capturing relevant features during the learning process. In our proposed model, it comprises two primary stages: squeezing and excitation, as shown in Fig. 2. In the squeezing stage, the network first employs a global average pooling layer where the mean value of every channel is calculated, resulting in a global context vector. This context vector is processed through two successive convolutional layers, with ReLU activation in between. The first convolutional layer reduces the channel by half, while the second restores it to the original size. Finally, a Sigmoid function is employed to output attention weights in the range between 0 and 1. In the excitation stage, a residual connection from the input goes to a dot product with those weights from the squeezing stage to modulate the importance of each channel in the original input.

**Bi-LSTM layer**. The Bi-LSTM layer aims to support, and comprehend the temporal dynamics and spatial hierarchies present in the PPG signals. The input features from the encoder are processed both forward and backward in the Bi-LSTM layer to identify long-term contextual characteristics and dependencies of the signals. Specifically, the PPG cycles exhibit temporal correlations and shared patterns in a given window. As a result, using Bi-LSTM here enables richer spatial-temporal feature representation in the latent space, improving the understanding of the signal during the transformation.

#### Discriminator

We further apply an adversarial training approach with the help of a discriminator model to enhance our model’s robustness. It is designed with the same architecture as the encoder from the proposed generator model architecture, except for one additional Dense layer at the end to output a single value. During adversarial training, the generator is trained to minimize the Hinge loss^52^, which is a widely used in adversarial training. It encourages the generator to produce PPG signals that are indistinguishable from reference signals by the discriminator. Simultaneously, the discriminator is trained to minimize the Hinge loss for real samples and maximize it for generated samples, leading to a more robust discriminator that can better distinguish between real and generated data. The Hinge loss function used by the discriminator is:

1 $$\begin{aligned} L_D = \frac{1}{N} \sum _{i=0}^{N} [\max (0, 1 - D(x_i)) + \max (0, 1 + D(z_i))], \end{aligned}$$

where $N$ is the number of windows, $x_i$ is the reference signal, and $z_i = G(y_i)$ is the enhanced signal, with $y_i$ as the original signal. $D(x_i)$ and $D(z_i)$ are the discriminator’s predictions for the reference and enhanced signals, respectively.

#### Custom loss function

In this section, we introduce a custom loss function designed for our task. Existing approaches for reconstructing corrupted signals have primarily relied on mean squared error (MSE) loss. However, due to the high sensitivity of PPG signals to environmental factors and amplitude variations, models trained with MSE alone often become unstable and struggle with generalization^53^. Moreover, a key limitation of MSE is that it treats all errors uniformly, regardless of their position within the signal. This is problematic because certain components of the PPG signal, such as the systolic peak, dicrotic notch, and diastolic peak, are more critical than others. To address this, we propose a custom loss function that incorporates PPG-specific domain knowledge, as shown in Formula 2.

2 $$\begin{aligned} L_C = {\left\{ \begin{array}{ll} MSE(z_i, x_i) \cdot \alpha + MSE(z_i, x_i) & \text {if } \alpha \ne 0, \\ L_{P2P}(z_i, x_i) \cdot \beta + MSE(z_i, x_i) & \text {if } \alpha = 0 \end{array}\right. } \end{aligned}$$

Specifically, based on the premise that an ideal PPG cycle is characterized by two peaks (systolic and diastolic peak) and one notch (dicrotic notch) between them, we introduce a variable, $\alpha$, which represents the difference in the number of peaks between the estimated and reference signals. Then, our custom loss function operates in two stages: 1) In the early stages of training, when the model often predicts PPG signals with an incorrect number of peaks ($\alpha \ne 0$), we use an additional term to penalize this by applying a factor proportional to $\alpha$ to the MSE loss. *This encourages the model to produce PPG cycles with the correct number of peaks (i.e., 2)*. 2) As training progresses and the PPG cycles approach the correct number of peaks (i.e., $\alpha =0$), we introduce an additional point-to-point loss ($L_{P2P}$) term scaled by a factor $\beta$ (0.01 empirically) over the MSE loss. Concretely, $L_{P2P}$ is calculated as the amplitude difference of the three points (two peaks and one notch) between the estimated and reference signals, which *encourages the model to refine the relative amplitude of the estimated signal*. Guided by this custom loss function, the model is better equipped to predict accurate and ideal PPG waveforms. Eventually, we define the generator loss as the combination of custom loss and generator-side Hinge loss, as follows:

3 $$\begin{aligned} L_G = \frac{1}{N} \sum _{i=0}^{N} L_C - \theta \cdot \frac{1}{N} \sum _{i=0}^{N} D(z_i), \end{aligned}$$

where $\theta$ is a hyper-parameter that is set to be 0.01 empirically and *N* denotes the total number of windows. We tested different $\beta$ and $\theta$ values and selected the best combination ($\beta$ = 0.01, $\theta$ = 0.01) for model training.

#### Model training

CP-PPG was implemented in PyTorch and trained on a single NVIDIA GeForce RTX 3090 GPU. The AdamW optimizer with $\beta _1 = 0.9$, $\beta _2 = 0.999$ was utilized for training. The initial learning rate was set to 0.001 and a learning rate scheduler with an *l*2 regularization term with a scale of 0.005 was applied. We limited the maximum training epoch to 300 and implemented the early stopping mechanism on the validation set to prevent model overfitting.

## Evaluation

To evaluate CP-PPG, we conducted comprehensive experiments as follows: 1) First, using the WF-PPG test dataset, we assessed the performance of the deep learning model from a morphological perspective by examining various waveform features. 2) Subsequently, we examined the effectiveness of the pre-trained model on four typical PPG downstream tasks by comparing the sensing performance obtained with PPG signals before and after the model transformation, on both the WF-PPG dataset and four public PPG datasets to demonstrate the generalizability. 3) Finally, we designed a new prototype in the form of a wristwatch and conducted a longitudinal in-the-wild test to validate the effectiveness of CP-PPG in real-life scenarios.

### Waveform transforming performance

#### Metrics

We used several metrics to evaluate the transformation performance from different perspectives. First, we considered three **overall metrics**: Mean Absolute Error (MAE), Dynamic Time Warping (DTW), and Pearson Correlation Coefficient (PCC). MAE measures the average error magnitude between the enhanced wrist PPG and finger PPG signals, reflecting overall accuracy. DTW accounts for temporal variations and optimally aligns the signals, offering a detailed comparison of their morphological similarity. PCC indicates the strength of correlation between the enhanced wrist PPG and reference finger PPG, with higher values showing greater waveform similarity.

**Fig. 3:**
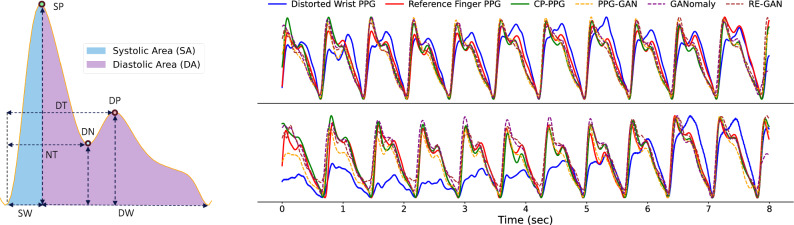
(Left) PPG point-based and area-based features used for CP-PPG ’s evaluation. (Right) Two examples illustrate the effectiveness of CP-PPG in refining the distorted wrist PPG signal.

Then, we further investigate detailed PPG waveform features, including point-based, area-based, and signal quality index (SQI)-based metrics. As shown in Fig. 3 (left), an ideal PPG cycle comprises three essential fiducial points: the systolic peak, diastolic peak, and dicrotic notch situated between them with their amplitudes and time scales are considered crucial features for quantifying the morphology of a PPG waveform and hold significant clinical importance. Thus, we considered **point-based metrics**^54,55^ including: the amplitude of the systolic peak (SP), time span from onset to the systolic peak (SW), time span from systolic peak to the next onset (DW); the amplitude of the dicrotic notch (DN), time span from onset to the dicrotic notch (NT); the amplitude of the diastolic peak (DP), time span from onset to the diastolic peak (DT). We utilized the mean absolute percentage error (MAPE) between the enhanced and reference PPG signals to demonstrate the transformation performance.

In addition to point-based metrics, areas under the curve of the signal also provide valuable insights into vascular dynamics^55^. Therefore, we consider two **area-based features**: systolic area (SA) and diastolic area (DA), which are defined based on the position of the systolic peak. We also used MAPE for area-based metrics.

Finally, we investigate CP-PPG ’s effectiveness using **signal quality indexes** (SQIs) including skewness and kurtosis, which are commonly used due to their ability to provide valuable insights into the shape and distribution of PPG waveform^56^. Specifically, in typical PPG signals, a slightly positive skewness is common, as the systolic peak is usually more pronounced, suggesting normal blood flow dynamics. Similarly, kurtosis measures the peakness or tailedness of a distribution, reflecting the shape of the waveforms and providing insights into their asymmetry. PPG signals often exhibit moderate kurtosis, reflecting the sharp systolic peak characteristic of normal waveforms. Here, we report skewness and kurtosis errors as Mean Absolute Error (MAE).

#### Baselines

In recent years, researchers have explored several generative methods for PPG signal reconstruction using deep neural networks. For example, recent approaches include convolutional autoencoders (AE-PPG)^57^, which learn to denoise motion-contaminated signals, and FC-GAN^58^, a GAN-based model that performs PPG reconstruction using only linear layers in both generator and discriminator. Another work, PPG-GAN^59^ employs a traditional Pix2Pix-inspired network^60^ with a U-Net-based generator to reconstruct motion-distorted PPG signals. Building upon this, RE-GAN^61^ integrates a CycleGAN-based generator^62^ with a Pix2Pix-based discriminator, further enhanced by a recursive loss to ensure consistency in the generated signals. Another approach^63^ adopts the GANomaly-based model, incorporating an additional latent space loss to align the embeddings of the generated and reference signals, thereby improving reconstruction performance. These methods primarily address motion artifacts during the reconstruction phase and aim to recover coarse-grained PPG waveforms (e.g., restoring the systolic peak). In contrast, CP-PPG focuses on reconstructing the fine-grained morphology of PPG signals, preserving all key features in the ideal morphology. We compare our method against the three aforementioned approaches, using the same dataset for both training and inference.

#### Results

**Table 1:** Waveform transformation performance with overall metrics (MAE, DTW, PCC), point-based metrics (MAPE %), area-based metrics (MAPE %), and SQI-based metrics (MAE).

	Overall Metrics			Point-based Metrics							Area-based Metrics		SQI-based Metrics	
	MAE	DTW	PCC	SP	SW	DW	DN	NT	DP	DT	SA	DA	Skewness	Kurtosis
Distorted	0.15	0.14	0.77	19.92	37.98	8.90	25.83	8.19	32.85	10.32	49.02	19.23	0.42	0.14
AE-PPG^57^	0.14	0.17	0.81	18.78	26.65	5.00	16.24	7.40	27.35	11.63	31.14	20.44	0.29	0.13
FC-GAN^58^	0.12	0.14	0.83	14.72	22.14	4.69	14.31	9.27	25.88	14.11	29.76	19.76	0.24	0.15
PPG-GAN^59^	0.10	0.09	0.89	10.21	20.96	5.01	13.35	10.01	16.45	12.08	25.08	16.23	0.27	0.13
GANomaly^63^	0.11	0.10	0.87	17.40	18.23	4.63	14.48	13.21	18.29	11.67	21.75	20.08	0.22	0.13
RE-GAN^61^	0.10	0.10	0.88	11.92	17.47	4.37	15.21	11.10	20.52	9.87	22.32	13.97	0.24	0.12
**CP-PPG**	**0.09**	**0.06**	**0.91**	**6.82**	**16.68**	**4.08**	**10.03**	**5.51**	**11.32**	**6.74**	**13.59**	**9.21**	**0.19**	**0.12**

*Waveform Illustration*: Fig. 3 (right) illustrates two examples of the distorted wrist PPG, reference finger PPG, and enhanced wrist PPG generated by three baselines and CP-PPG. Overall, the distorted wrist PPG signals are transformed to closely resemble the ideal morphology of the reference finger PPG signals, when using CP-PPG. This superior performance can be attributed to CP-PPG ’s unique design, which incorporates specialized modeling components, such as the custom PPG-aware loss function. Notably, we found that 96.12% of PPG cycles lacking the diastolic peak were successfully recovered by CP-PPG. In contrast, while the three baselines also restore the coarse-grained morphology, they perform poorly in recovering the diastolic peaks and dicrotic notches, which are essential for certain downstream tasks.

*Quantitative Results: * Table 1 presents a quantitative comparison of CP-PPG with other baselines on the aforementioned metrics. The results clearly show that CP-PPG achieves significantly lower errors across all metrics compared to the distorted case, demonstrating its ability to accurately recover and refine the location and amplitude of the three essential fiducial points. While the baselines also show some improvement, their performance remains consistently inferior to CP-PPG, which excels in handling the CP variations that the baselines are not specifically designed to address.

These quantitative analyses, complemented by visual comparisons (Fig. 3), highlight the effectiveness of CP-PPG in rectifying distorted wrist PPG signals, aligning them more closely with the ideal characteristics of finger PPG signals, thereby potentially enhancing the sensing performance of downstream physiological tasks.

### Ablation studies

In this section, we evaluate the impact of different model configurations on transformation performance to gain insight into how CP-PPG achieves its results.

#### Influence of key model components

To assess the contribution of different model components, including the PPG blocks, Bi-LSTM layers, custom loss function, and adversarial training scheme, we systematically removed one component at a time from the default proposed model. Specifically, we started by eliminating the adversarial training scheme, which removes the discriminator and trains the model with the generator only. Subsequently, the proposed custom loss function was removed and replaced by normal MSE loss. Next, we took the Bi-LSTM layer away to assess its effectiveness in capturing long-term dependencies in the model’s latent space. Lastly, by replacing the designed PPG blocks with standard CNN blocks of the same depths, we examined a normal 1D CNN encode-decode model.

**Table 2:** Ablation studies. 1) Influence of key model components, including $\textcircled {1}$ PPG blocks, $\textcircled {2}$ Bi-LSTM, $\textcircled {3}$ Custom loss, $\textcircled {4}$ Adversarial training; 2) Effect of model depth (MD); and 3) Effect of window length (WL).

1. Influence of key model components																	
Components				Overall Metrics			Point-based Metrics							Area-based Metrics		SQI-based Metrics	
$\textcircled {1}$	$\textcircled {2}$	$\textcircled {3}$	$\textcircled {4}$	MAE	DTW	PCC	SP	SW	DW	DN	NT	DP	DT	SA	DA	Skewness	Kurtosis
$\checkmark$	$\checkmark$	$\checkmark$	$\checkmark$	**0.09**	**0.06**	**0.91**	**6.72**	**16.68**	**4.08**	**10.03**	**5.51**	**11.32**	**6.74**	**13.59**	**9.21**	**0.19**	**0.12**
$\checkmark$	$\checkmark$	$\checkmark$		0.09	0.07	0.90	6.82	16.11	4.37	11.35	5.37	12.09	6.81	15.61	12.84	0.25	0.16
$\checkmark$	$\checkmark$			0.10	0.08	0.88	6.88	18.04	4.48	11.31	6.15	12.56	8.20	17.51	16.37	0.33	0.19
$\checkmark$				0.11	0.08	0.87	6.92	19.35	4.86	12.81	6.39	14.58	8.20	21.73	17.01	0.35	0.19
				0.13	0.09	0.85	8.36	18.54	4.63	20.70	6.55	22.48	8.96	19.68	22.33	0.39	0.24

**2. Effect of model depth**																	
MD		#Params		Overall Metrics			Point-based Metrics							Area-based Metrics		SQI-based Metrics	
				MAE	DTW	PCC	SP	SW	DW	DN	NT	DP	DT	SA	DA	Skewness	Kurtosis
1		80K		0.11	0.08	0.89	8.15	17.11	4.93	11.90	6.61	12.03	7.73	14.31	14.04	0.27	0.13
3		1.1M		**0.09**	**0.06**	**0.91**	**6.82**	**16.68**	**4.08**	**10.03**	**5.51**	**11.32**	**6.74**	**13.59**	**9.21**	**0.19**	**0.12**
5		17.5M		0.08	0.06	0.91	7.48	15.62	3.79	9.77	5.39	11.48	7.36	12.16	9.48	0.20	0.12

3. Effect of input window length																	
WL				Overall Metrics			Point-based Metrics							Area-based Metrics		SQI-based Metrics	
Train		Test		MAE	DTW	PCC	SP	SW	DW	DN	NT	DP	DT	SA	DA	Skewness	Kurtosis
3s		3s		0.12	0.12	0.85	8.41	17.53	4.26	13.14	6.84	17.79	8.04	17.22	11.15	0.22	0.16
5s		5s		0.12	0.09	0.87	7.15	16.79	4.07	11.05	6.47	14.26	7.07	16.93	11.49	0.20	0.14
**8s**		**8s**		**0.09**	**0.06**	**0.91**	**6.82**	**16.68**	**4.08**	**10.03**	**5.51**	**11.32**	**6.74**	**13.59**	**9.21**	**0.19**	**0.12**
8s		3s		0.11	0.12	0.87	7.89	18.40	4.16	13.04	7.01	16.37	8.74	17.43	11.60	0.23	0.16
8s		5s		0.10	0.08	0.89	7.01	17.19	4.13	11.13	6.06	13.39	7.05	15.50	9.95	0.20	0.14

Table 2 displays the settings and their corresponding performance in overall metrics and our predefined feature-based metrics. We can observe that the transformation performance gradually decreases with the removal of each additional component, suggesting that every component positively contributes to the model. Notably, the MAPEs of the diastolic-related metrics suffer from a significant increase after removing the custom loss function and adversarial training setting, which in turn demonstrates their crucial roles in the model. This is attributed to the special design of the custom loss, which encapsulates the intricate domain knowledge inherent in PPG waveforms, compelling the model to prioritize the accurate restoration of critical features like the diastolic peak. Meanwhile, in adversarial training, the discriminator robustly learns to distinguish between the enhanced and reference signals, in a way that guides the generator toward producing outputs that more closely resemble authentic signals.

#### Impact of model depth (MD)

The PPG block, designed to capture the intricate characteristics of the PPG waveform, has proven to be highly effective in improving the transformation performance of CP-PPG, as evidenced in Table 2. In the default model, we stacked three PPG blocks in the encoder and decoder, respectively. Since the model depth ( i.e., the number of PPG blocks) directly influences the model’s capacity, we evaluate its impact on the transformation performance. Specifically, we trained two additional models with *MD*=1 and *MD*=5 respectively, and compared their performance with the default model (*MD*=3).

As shown in Table 2, the transformation performance tends to increase with the model depth, attributed to the increased model capacity. However, we observed a marginal improvement in performance or even degradation on specific features (e.g., SP, DT) when *MD* increases from 3 to 5. This could be attributed to the model with $MD=3$ already capturing the correlation between waveform transformation, and deeper models might lead to overfitting. The model with $MD=5$ (17.5M parameters) is also approximately 16 times larger than that with $MD=3$ (1.1M parameters), significantly increasing the system overhead (e.g., latency and power consumption) of training and inference. Therefore, we selected $MD=3$ as the default setting as it strikes an optimal balance between transformation performance and system overhead.

#### Impact of input window length (WL)

In the default configuration, we segmented the PPG signals into eight-second windows for model training and testing. As the PPG signal presents strong temporal correlations, the length of the input window might affect the transformation performance. Thus, for this ablation study, we prepared two additional versions by segmenting the PPG signals into windows with lengths of three seconds and five seconds, respectively, which are then utilized to train the corresponding models. Notably, this experiment does not extend to longer window lengths as it results in fewer windows, which can weaken our model training.

From Table 2, we can observe that with the increase in window length, the transformation performance consistently improves across most predefined metrics, suggesting that a larger input window length allows the model to capture the temporal dependencies more accurately. Next, given that our generator with autoencoder-based architecture can accommodate inputs of varying lengths, we further tested the eight-second model using data with three-second and five-second window sets (i.e., only perform inference). Remarkably, the eight-second model still outperformed the other two models, regardless of the input window length during testing. This finding underscores the versatility of our model, which can be effectively deployed across diverse practical scenarios to accommodate different window sizes.

### Performance on physiological tasks

#### Experiment setup

*While the above evaluations demonstrated the effectiveness of CP-PPG in refining the morphology of PPG waveforms towards ideal ones, it remains uncertain whether the refined waveforms can improve PPG sensing performance and to what extent.* Therefore, we selected four typical PPG downstream tasks - HR estimation, heart rate variability (HRV) estimation, BP estimation, and respiratory rate (RR) estimation - to assess the effectiveness of CP-PPG. Specifically, for a given dataset of the same task, we would compare the PPG signals before and after being quality-enhanced by the pre-trained CP-PPG (as API). It is important to note that we applied typical signal processing for both cases (e.g., filtering, DC removal, etc.) and segmented the signal into windows with the length required for the task, before doing the same task-specific analysis to compare final results.

#### Additional datasets

We utilized both the WF-PPG dataset (test) and other public datasets to evaluate CP-PPG ’s performance on different downstream tasks. In the WF-PPG dataset, we took the ECG signal alongside PPG signals, which enables the derivation of the ground truth for HR and HRV. The majority of other PPG datasets in the public domain were acquired during various intensive activities such as walking and running, as they aim to investigate the impact of motion artifacts. On the other hand, since CP-PPG is designed to address signal distortion resulting from changes in tightness under sedentary conditions, our primary criterion for selecting public datasets was that they should contain PPG data collected in stationary or sedentary scenarios. Following that, we selected four datasets to evaluate different downstream tasks described below.

**PTT-PPG**^45^: this dataset mainly consists of three data streams, including one ECG and two reflectance PPGs, one from the fingertip and the other from the base of the finger, from 22 healthy subjects while still, walking, and running. We only used the data recorded during stationary periods and enhanced the finger base PPG using pre-trained CP-PPG to evaluate its performance on HR and HRV estimation.

**PPG-DaLiA**^46^: this dataset was recorded with a wrist-worn smartwatch Empatica E4 (provides wrist PPG signal) and a chest-worn device RespiBAN (offers ground truth data for HR and HRV derived from ECG) on 15 subjects. Therefore, we utilized it to evaluate HR and HRV estimation. Similarly to the PTT-PPG dataset, PPG-DaLiA was collected under various activities and we only used the data recorded during stationary periods for our evaluation.

**WESAD**^64^: similar to PPG-DaLiA, the WESAD dataset also contains wrist PPG signals along with ground truth data for HR and HRV from ECG signals, and RR from respiration signals. The data was collected from 15 participants engaging in sedentary activities such as sitting, watching, and speaking. Thus, we utilized most of the data for the evaluation of HR estimation, HRV estimation, and RR estimation.

**Graphene-HGCPT**^65^: this dataset comprises a PPG signal recorded with a wrist-worn sensor alongside corresponding continuous arterial blood pressure (ABP) recordings obtained using the medical-grade Finapres NOVA device’s finger cuff that estimates brachial BP. The data was collected from 7 young, healthy individuals, each underwent a series of lower motion activities to intentionally elevate blood pressure levels. We utilized this data to evaluate both Systolic BP (SBP) and Diastolic BP (DBP) estimation.

#### Heart rate and heart rate variability estimation

We estimated HR in beats per minute (bpm) based on the number of detected pulse **valleys** (PPG) in the processed windows (8s), and used Mean Absolute Error (MAE, in bpm) to assess estimation performance. HRV metrics were calculated from the time differences between consecutive pulse **valleys**^66,67^. We selected two typical metrics, including Root Mean Square of the Successive Differences (RMSSD, in ms), and Standard Deviation of R-R Intervals (SDRR, in ms) to quantify HRV estimation accuracy.

From Table 3, we can observe that across all datasets, the enhanced PPG signal consistently exhibits lower errors in all metrics for both HR (21.96% on average) and HRV estimation (45.70% in RMSSD and 40.53% in SDRR). This improvement can be attributed to CP-PPG ’s ability to compensate for temporal shifts induced by variations in contact pressure during the morphology refinement process. By refining the morphology, the enhanced signal provides more accurate heartbeat timing information, resulting in enhanced HR and HRV estimation accuracy. The WF-PPG dataset obtains lower performance yet larger improvement as it was collected under more diverse CP levels, while the CP in other public datasets is relatively stable.

#### Respiratory rate estimation

We adopted a deep learning approach as introduced by Osathitporn et. al.^8^. Specifically, we employed five-fold cross-validation, ensuring that test folds contain different subjects and cover all subjects from the processed dataset. Both the original (distorted) and enhanced signals were segmented as 16*s* windows and then trained under the same model architecture. Here, following prior work^8^, 16*s* window length is selected due to the relatively slower cyclic nature of respiratory signals (12 to 20 breaths per minute), ensuring a reliable estimation with a larger window size. The results are reported as the average and standard deviation of the testing MAE in breaths per minute (brpm) across five folds, as shown in Table 3. Evidently, using enhanced signals notably outperformed the distorted signals by 6.85%. The reason is that during the morphology transformation process, CP-PPG can regulate the amplitude shifts caused by changes in contact pressure. As a result, the rhythm information, which is crucial for RR estimation, is better preserved in the enhanced signal.

#### Blood pressure estimation

For BP estimation, we followed the commonly used machine learning-based approaches for SBP and DBP prediction (mmHg)^55,68,69^. In detail, we extracted multiple PPG waveform features such as SP, SW, and DW, along with other features such as HR and HRV, from the PPG windows (8s). These features were then utilized to train an Adaboost model for BP estimation. However, since many distorted PPG signals lack the diastolic peak, we could only extract a limited set of waveform features, including: SP, pulse area (PA), PA/SP, SW, DW, mean R-R intervals, SDRR, RMSSD, the percentage of successive R-R intervals differing by more than 50 ms (PNN50), and High Frequency (HF) power in spectral analysis. On the other hand, thanks to CP-PPG ’s ability to restore the diastolic peak and dicrotic notch, we were able to extract five additional waveform features: DN, NT, DP, DT, DT-SW.

**Table 3:** Physiological estimation performance with and without our model transformation across all datasets (MAE).

Dataset	Size (min)	PPG Input	HR (bpm)	HRV		RR (brpm)	BP	
				RMSSD (ms)	SDRR (ms)		SBP (mmHg)	DBP (mmHg)
WF-PPG (test)	528	Distorted	1.84 ± 1.17	52.05 ± 40.89	31.03 ± 22.68	–	–	–
		Enhanced	**1.12** ± **1.00**	**9.43** ± **6.59**	**8.04** ± **4.78**	–	–	–
PTT-PPG	399	Distorted	0.54 ± 0.18	5.37 ± 1.70	3.58 ± 1.20	–	–	–
		Enhanced	**0.50** ± **0.12**	**4.97** ± **1.15**	**3.28** ± **1.04**	–	–	–
PPG-DaLiA	554	Distorted	1.01 ± 0.32	17.79 ± 6.18	13.87 ± 4.65	–	–	–
		Enhanced	**0.88** ± **0.28**	**11.20** ± **3.28**	**9.91** ± **3.13**	–	–	–
WESAD	2238	Distorted	1.09 ± 0.11	26.97 ± 12.21	17.79 ± 6.49	2.92 ± 0.43	–	–
		Enhanced	**0.78** ± **0.09**	**12.29** ± **2.86**	**9.05** ± **1.90**	**2.72** ± **0.35**	–	–
Graphene-HGCPT	753	Distorted	–	–	–	–	7.00 ± 0.26	4.63 ± 0.14
		Enhanced	–	–	–	–	**6.65** ± **0.20**	**4.46** ± **0.15**

Table 3 displays testing MAE average and standard deviation for SBP and DBP across five folds. We can observe that the enhanced PPG signals with refined typical features and additional diastolic features provide better predictions (5% in SBP, and 3.67% in DBP) of blood pressure compared to using the distorted PPG (only typical features). This improvement can be attributed to the enhanced PPG signals having more accurate physiological characteristics, which facilitate more accurate feature extraction used for training the model.

### Performance in In-the-wild settings

#### Experiment setup

The above evaluations were conducted under controlled conditions and may not fully represent real-world scenarios. Specifically, in the training dataset, the contact pressure of the wrist PPG sensor is manually adjusted using a clamp device^39^, which does not accurately reflect the *natural* variations in contact pressure caused by posture changes in real-life situations. Additionally, public datasets were typically collected in confined environments, where subjects are instructed to perform specific activities or maintain certain states^45,46,64,65^.

**Fig. 4:**
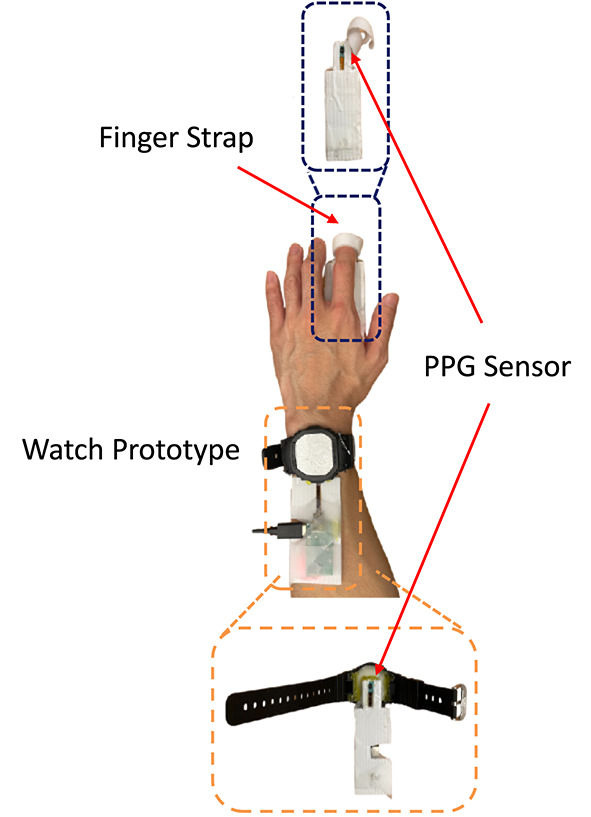
Illustration of our watch prototype for in-the-wild study.

To address these issues, we conducted a longitudinal in-the-wild study with five participants over five consecutive days. First, to ensure user comfort during the experiments, we present a smartwatch-based prototype, as shown in Fig. 4. Specifically, we embedded the wrist PPG sensor at the bottom of an empty watch case, allowing participants to wear it with an adjustable strap for their preferred tightness. The finger PPG sensor was secured with a clip, and participants also wore an ECG chest band to obtain ground truth data for HR and HRV.

**Table 4:** HR/HRV performance on in-the-wild study (MAE).

	PPG Input	HR (bpm)	HRV	
			RMSSD (ms)	SDRR (ms)
Subject 1	Distorted	1.47 ± 0.31	15.10 ± 3.24	12.44 ± 3.80
	Enhanced	**0.52** ± **0.11**	**5.21** ± **1.13**	**6.65** ± **2.90**
	Reference	0.51 ± 0.10	5.13 ± 1.04	6.26 ± 2.44
Subject 2	Distorted	1.09 ± 0.41	11.14 ± 4.26	11.03 ± 3.50
	Enhanced	**0.87** ± **0.26**	**8.82** ± **2.70**	**10.18** ± **2.88**
	Reference	0.94 ± 0.33	9.58 ± 3.33	10.64 ± 3.11
Subject 3	Distorted	1.55 ± 0.58	16.08 ± 5.97	18.12 ± 5.51
	Enhanced	**1.26** ± **0.62**	**13.18** ± **6.42**	**17.13** ± **5.66**
	Reference	1.29 ± 0.64	13.45 ± 6.66	17.03 ± 5.65
Subject 4	Distorted	0.57 ± 0.23	5.89 ± 2.44	6.79 ± 1.33
	Enhanced	**0.36** ± **0.06**	**3.70** ± **0.57**	**6.33** ± **0.92**
	Reference	0.32 ± 0.07	3.30 ± 0.68	6.21 ± 0.64
Subject 5	Distorted	1.57 ± 0.58	16.00 ± 6.09	15.43 ± 2.75
	Enhanced	**0.89** ± **0.16**	**8.95** ± **1.59**	**12.28** ± **2.78**
	Reference	0.86 ± 0.20	8.51 ± 1.96	11.42 ± 3.04

Each participant wore the devices for 30 minutes daily, during which they were free to participate in any desk-related sedentary activities such as working on a computer, reading a book, watching videos on the phone, and sitting on a chair. These activities naturally led to posture changes, causing varying degrees of morphology distortion in the wrist PPG signal. After collecting the data, we applied the previous evaluation pipeline for downstream tasks to the wrist PPG signal (distorted and enhanced) to evaluate the HR and HRV performance, using the ground truth from ECGs. Similarly, we also evaluated the HR and HRV results of the finger PPG signal (denoted as Reference), which serves as a reference performance achievable using finger-based PPG devices (e.g. pulse oximeter).

#### Performance on HR and HRV estimation

Table 4 compares the HR and HRV estimation performance (MAE) among the distorted wrist PPG signals, enhanced wrist PPG signals with CP-PPG, and the finger PPG signals (reference) from our in-the-wild data. The values are averaged over all the data collected during the five days. We can observe that: 1) for all the HR and HRV metrics, the enhanced wrist PPG signal significantly outperforms the distorted signal, and the performance is very close to that of the finger PPG signal. This is because the data collected with the developed clamp prototype under various CPs effectively captures comprehensive variations of the PPG morphology to operate in realistic conditions; 2) the results are consistent across different subjects, indicating the robustness and generalizability of our approach; 3) an interesting observation is that the enhanced signal can even outperform the reference signal in some cases (e.g., Subject 2). The reason is that the reference signal is from a finger PPG, which can still suffer from poorer contact pressure depending on the behaviors of the users despite an initial perfect fit, leading to degraded performance.

### System performance

**Table 5:** System performance of CP-PPG.

	Memory	Latency	Energy
	(MB)	(ms)	(J/sample)
MacBook Air M1	85.85	11.65	–
Raspberry Pi 5	174.36	69.74	0.29
Xiaomi 13 Phone	127.30	60.12	0.11

Since CP-PPG can be used as a plug-in API, we investigate its system performance regarding peak memory usage, latency, and energy consumption. The measurements were carried out on three devices: a MacBook Air M1, a Raspberry Pi 5, and a Xiaomi 13 smartphone. Table 5 presents the results averaged over 100 inferences. We can observe that CP-PPG operates efficiently, with peak memory usage remaining under 180 MB across three devices, which translates to only 2% of the total memory budget even on the Raspberry Pi 5. The Macbook Air exhibited the lowest memory usage, likely due to its optimized memory management and superior hardware architecture. In terms of latency, CP-PPG processes an eight-second PPG sample in under 100 ms on all platforms, indicating its capability for real-time inference. Additionally, CP-PPG is energy-efficient; for instance, on the Xiaomi 13 smartphone, it consumes only 0.11 J, a negligible fraction of the overall battery capacity (71,280 J). These results demonstrate that CP-PPG is a lightweight model, making it well-suited for deployment on typical edge-scale devices.

## Discussion

Our CP-PPG addresses detrimental variations in contact pressure between the skin and sensor during static hand postures in sedentary activities. For example, reading a book may result in a still but bent wrist, which alters the degree of contact pressure and affects signal quality. While some studies have revealed the impact of CP, to the best of our knowledge, no existing work has proposed a solution to mitigate its effects. CP-PPG presents a novel morphology-guided PPG waveform restoration network and a domain-specific loss function. Our extensive experiments on diverse PPG datasets and downstream tasks demonstrate the effectiveness and generalizability of CP-PPG in improving PPG sensing performance. Building on this, we outline several potential discussions where CP-PPG may inform broader PPG signal enhancement as follows:

*Adaptation to motion artifacts*: Motion artifacts (MAs), a central focus in existing studies, share certain interdependencies with CP. Consequently, CP-PPG may also inform and inspire future research on mitigating MAs in PPG signals. Specifically, MAs typically arise from substantial physical movements of both the human body and the PPG sensor, such as wrist and watch motion during walking and running. These movements can cause misalignment between the sensor and skin (parallel to the skin surface) or dynamic fluctuations in CP (perpendicular to the skin surface), both of which distort the PPG signal. In other words, while MAs occur exclusively during motion states involving actual body movement, both sedentary and motion states can influence CP. Consequently, CP may serve as a complementary modality to acceleration for mitigating the effects of MAs, opening a promising direction for future research on MA reduction.

*Demographic considerations: *In this work, as a first step into addressing CP’s effects on PPG measurement, we chose training data from young, healthy participants who generally present the ideal PPG morphology. However, even with optimal contact pressure, the PPG morphology of older individuals (due to vascular aging^70^) or those with certain cardiovascular diseases^71^) (e.g., arrhythmias) deviates from this ideal morphology. Consequently, the trained model in this work may not be directly applicable to these populations. However, the proposed framework remains valuable. Future researchers can collect appropriate data for these demographic groups following our experimental conditions, and fine-tune the model considering that the finger PPG signal under optimal contact pressure may not present the ideal morphology.

*Multiple PPG channels:* PPG sensors can also operate in different color channels, including green, red, and infrared, with each color penetrating to different depths and measuring different types of blood vessels^72^. We focus on transforming PPG signals from the green light channel, as it is typically used in smartwatch settings. However, the proposed framework can also be applied to other color channels, where PPG sensors might be deployed in different measurement sites (e.g., ear). Moreover, given that different colors exhibit distinct optical characteristics, it is possible to leverage the inherent correlation of PPG waveforms across different color channels to enhance the performance and reliability of waveform transformation. For instance, if the PPG waveform from the red channel is corrupted due to real-world factors, the other channels can still facilitate the transformation process. Future works may consider integrating features from multiple PPG channels to generate an even more robust, improved signal.

## Related work

### Impact of contact pressure

Recent works have begun to analyze the impact of contact pressure between human skin and the sensor on the PPG signal^34,49,73^. Specifically, as human skin is soft, varying contact pressures cause different degrees of tissue deformation and thus varying lengths of light propagation. Consequently, the signal quality of the PPG signal is affected. For example, May et al. experimentally demonstrated the correlation between contact pressure and the amplitudes of AC and DC components, indicating the existence of an optimal contact pressure that generates PPG signals with the highest signal-to-noise ratio (i.e., ideal morphology)^74^. In addition, Scardulla et al. investigated the impact of contact pressure on the HR estimation accuracy at various exercise intensities using a smartwatch-like device, finding that higher contact pressures enable more accurate heart rate estimation^75^. However, these works merely highlight the issue of contact pressure without connecting it to real-world scenarios and proposing solutions to address it. Our work delved deeper into the problem of contact pressure. It proposed a generic approach that can convert PPG signals measured at sub-optimal skin-sensor contact into ideal waveforms, demonstrating its effectiveness with comprehensive experiments.

### Combating motion artifacts

Motion artifacts are unavoidable in wearables like smartwatches, prompting numerous efforts to mitigate their effects. Conventional signal processing techniques have addressed motion-related distortions in PPG signals. For example, adaptive filtering methods, such as the adaptive step-size least mean squares filter^76^, utilize motion data from an accelerometer as a reference^31^ to remove these artifacts. Recently, deep learning techniques, including convolutional neural networks^77,78^, long and short-term memory^79^, generative adversarial-based networks^32,59,61,63,80^, have been employed for motion-distorted PPG signals, achieving downstream results that surpass traditional signal processing approaches.

Our work identifies PPG morphology variations caused by contact pressure changes during sedentary activities, an aspect often overlooked in previous research. Unlike prior waveform reconstruction efforts that primarily focus on recovering the systolic peak for HR estimation, our study emphasizes the reconstruction of finer-grained PPG waveform features. This approach aims to achieve an ideal PPG morphology, enabling more advanced downstream applications that require sophisticated features.

## Conclusion

In this study, we introduced CP-PPG, a first framework designed to transform low-quality PPG waveforms measured under suboptimal contact pressure into ideal ones. CP-PPG comprises a suite of well-crafted signal processing techniques, and a lightweight deep-learning model with specialized designs. The extensive results demonstrated that CP-PPG not only enhances PPG signal quality but also improves sensing performance across four typical PPG downstream tasks. Notably, the performance on public PPG datasets and in-the-wild experiments highlights CP-PPG ’s versatility in transforming distorted PPG signals, showcasing its significance in achieving accurate and robust physiological sensing under realistic conditions.

## Data Availability

The study’s processed datasets are available from the corresponding author on reasonable request.
